# Intimate Partner Violence Correlates With A Higher HIV Incidence Among MSM: A 12-Month Prospective Cohort Study in Shenyang, China

**DOI:** 10.1038/s41598-018-21149-8

**Published:** 2018-02-13

**Authors:** Hong-yi Wang, Ning Wang, Zhen-xing Chu, Jing Zhang, Xiang Mao, Wen-qing Geng, Yong-jun Jiang, Hong Shang, Jun-jie Xu

**Affiliations:** 1 Key Laboratory of AIDS Immunology of the National Health and Family Planning Commission, Department of Laboratory Medicine, The First Affiliated Hospital, China Medical University, No. 155, Nanjingbei Street, Heping District, Shenyang, Liaoning Province 110001 China; 2 Collaborative Innovation Center for Diagnosis and Treatment of Infectious Diseases, Hangzhou, China; 3 National Center for AIDS/STD Control and Prevention, Chinese Center for Disease Control and Prevention, Beijing, 102206 China

## Abstract

Intimate partner violence (IPV) and HIV are highly prevalent worldwide among MSM. However, the association between IPV and HIV seroconversion is virtually unknown. This 12-month prospective cohort study was conducted among MSM in Shenyang, China to explore the causality between IPV and the incidence of HIV. Adjusted Hazard Ratios (aHRs) of HIV acquisition were derived from a multivariate time-dependent Cox model and applied to calculate population attributable fractions (PAFs). Among 476 HIV-negative MSM subjects, 89(18.7%) reported being victims of IPV in the past 3 months (P3M). IPV was significantly correlated with lower education, having more condomless anal intercourse (CAI) and being depressed (each P < 0.05). The incidence of HIV among IPV victims was 11.3/100 PY compared to 3.8/100 PY in non-IPV-victims. Furthermore, IPV victimization was independently associated with HIV seroconversion (aHR = 4.1, PAF = 37.9%). Other predictors for seroconversion included non-local residence in Liaoning province (aHR = 3.9, PAF = 45.2%), engaging in condomless receptive anal intercourse (CRAI)(aHR = 3.1, PAF = 24.2%) or CAI with casual male partners (aHR = 3.8, PAF = 26.3%) in the P3M and syphilis infection (aHR = 4.7, PAF = 33.7%) (each P < 0.05). IPV increased the HIV seroconversion risk of MSM, with a high PAF. HIV prevention programs should integrate IPV screening and intervention, and MSM affected by IPV need to be preferentially enrolled in pre-exposure prophylaxis.

## Introduction

Globally, men who have sex with men (MSM) population continue to disproportionately suffer from HIV^1–4^. MSM are also currently the group with fastest growth in the HIV epidemic occurring in China. The proportion of new HIV cases nationwide in China due to male homosexual contact each year has increased from 12.2% in 2007^5^ to 26.4% in 2016^6^. This indicates that some risk factors that fuel the rapid spread of HIV among Chinese MSM are still not well controlled.

HIV transmission events can occur in various social contexts, and therefore, social factors are core elements that may affect HIV vulnerability, transmission and the impact of behavioral prevention programs. However, few HIV prevention programs address these issues, especially for MSM, due to the very limited available data on these topics in comparison to the substantial number of investigations conducted regarding their risky sexual behavior^7–10^. Intimate partner violence (IPV) is one prominent social factor that is highly prevalent in many communities and is linked to more mortality and a higher burden of several other diseases among the general population. It is defined as the continuum from a single episode of violence to ongoing battering within a close relationship^11^. IPV has been associated with a higher prevalence of HIV, particularly among heterosexual females^12,13^. Recent work has hinted that MSM may have a similar or higher prevalence of IPV compared with the heterosexual population. The National Violence Against Women Survey (NVAWS) in the United States showed that the lifetime prevalence of physical IPV was 25% in MSM compared to 8% in heterosexual males and 21% in females^14^. Studies on MSM in Shanghai, China revealed that approximately 40% of MSM had experienced IPV during their lifetime^15,16^.

Although the causality between IPV and HIV infection has been well-established in the female population, this does not mean that it also applies equally to MSM. There is a distinctive difference in the sexual behaviors, physiology, and cultural features of MSM and heterosexual females. However, little is known globally about the effects of partner violence on the HIV epidemic in the MSM population. Literature on this topic mainly originates from cross-sectional studies conducted in developed Western countries, which observed associations between IPV and HIV high-risk practices (e.g., condomless anal intercourse [CAI], substance abuse) and the prevalence of HIV in MSM^17–24^. Two studies conducted in China found that MSM that report IPV were more likely to engage in CAI and had a higher prevalence of HIV^15,25^. However, the causality between IPV and HIV infection could not be established because of its cross-sectional study design. One single longitudinal study on MSM in the United States reported a long-term direct relationship between lifetime IPV and HIV seroconversion during a 5-year follow-up^26^. In most circumstances, IPV among MSM is a time-varying determinant that may change as a result of changes in partners or partner dynamics^27^. To understand the causality between IPV and HIV, it is more rational and feasible to shorten the monitoring interval of IPV and to analyze it as a time-dependent determinant by examining the correlation between a recent IPV experience and HIV seroconversion.

The contribution of a risk factor to a disease is usually quantified using a population attributable fraction (PAF). A PAF is interpreted as the proportional reduction in population disease risk that would occur if exposure to a specific risk factor were reduced to an alternative ideal exposure scenario. Hence, a risk factor with a higher PAF would require greater intervention. However, nothing is known about the PAF of IPV for the incidence of HIV in MSM, and this knowledge gap has made it more difficult to prioritize intervention strategies.

One important component in the implementation of a comprehensive HIV prevention strategy is to not only gain an understanding of the role of recent IPV victimization in HIV incidence for MSM but also to quantitatively assess the effects of IPV on new HIV infections. To that end, the purposes of this prospective cohort study were (1) to investigate the prevalence of IPV in a sample of MSM in Shenyang, China in order to explore possible relationships between IPV and other risk factors and the incidence of HIV in these men and (2) to estimate and compare the new HIV infection risk attributable to IPV and other risk factors by calculating PAFs.

## Results

### Baseline participant characteristics

**Figure 1:**
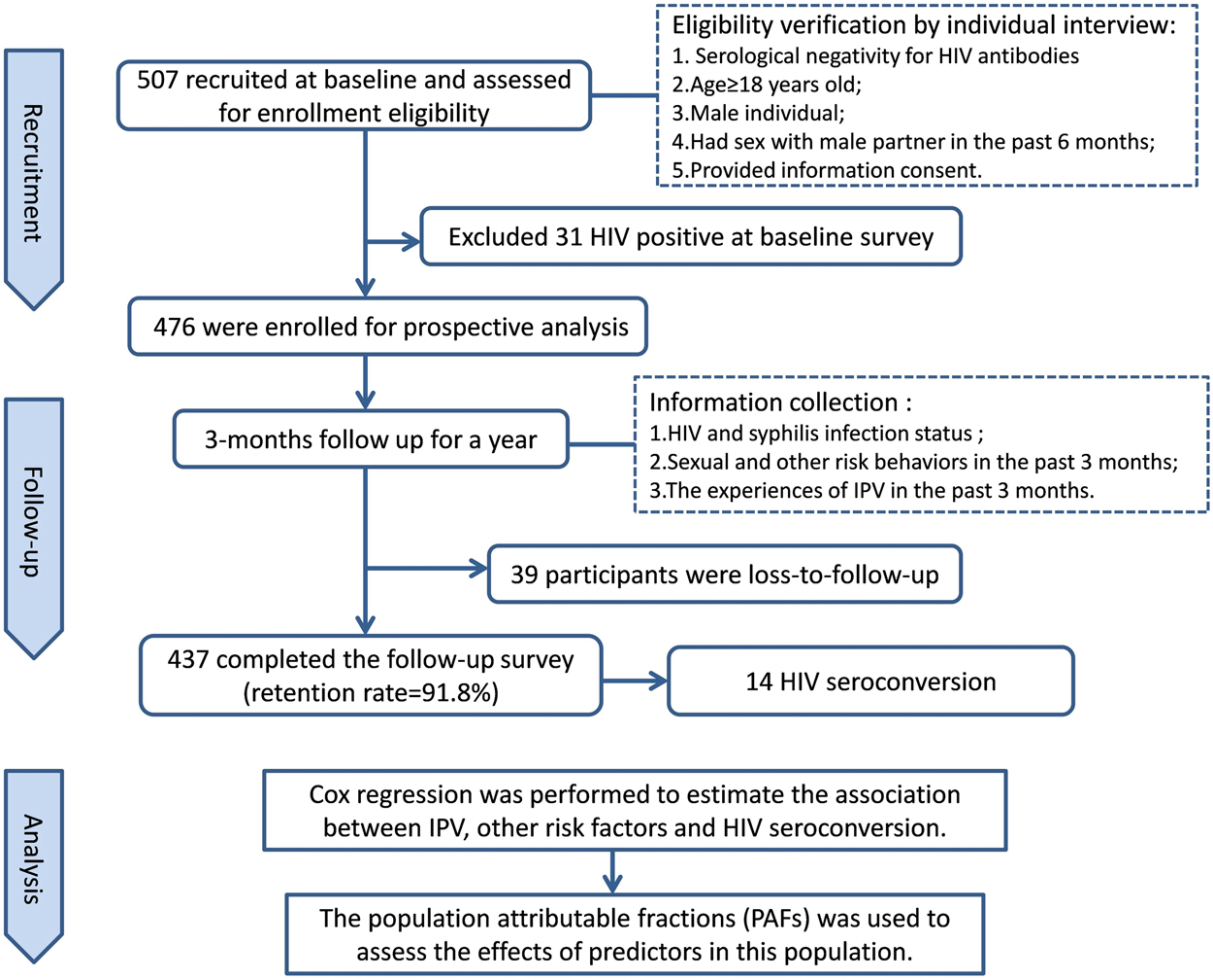
Participation of eligible men who have sex with men in the open cohort study in Shenyang, China.

As shown in Fig. 1, a total of 507 MSM attended our baseline-screening survey. Of these, 476 baseline HIV negative MSM were included in the longitudinal survey.

At baseline, eligible MSM subjects were relatively young, with a median age of 28.0 (IQR: 24.0–34.0) years old. Approximately two-fifths of the participants had received at least a college education, and 64.3% were single. Approximately 40% of participants reported having had multiple male casual male sex partners, 20.2% had CAI with male casual partners and 22.0% reported engaging in CRAI in past three months. The baseline seroprevalence of syphilis was 12.2%. The detection rate of symptoms of depression (CES-D score ≥ 15) was 37.2%.

### The prevalence of IPV and its associated factors

In the three months prior to enrollment, nearly one-fifth (18.7%) of our MSM participants reported that they had been subjected to IPV from male partners. Specifically, 9.9% had been a victim of physical IPV, 13.7% had been victimized by psychological IPV including threats and emotional abuse, and 9.5% were victims of sexual IPV (Table 1).

**Table 1:** Prevalence of IPV among MSM at baseline (N = 476).

Types of violence	No. of IPV victims	Prevalence
Physical IPV (e.g., Has someone hit you or thrown something at you?)	47	9.87%
Psychological IPV (Stalking and psychological aggression)	65	13.66%
Sexual IPV (Has someone forced you to have sex when you did not want to?)	45	9.45%
Any form of IPV in the P3M	89	18.70%

Abbreviations: IPV = intimate partner violence; P3M = past three months.

After adjusting for background variables including age, monthly income and marital status, MSM who had been victims of IPV in the P3M were more likely to be less-educated (high school and below) (aOR = 1.8, 95%CI = 1.1–3.0), to have CAI in the P3M (CIAI with male partners: aOR = 1.8, 95%CI = 1.1–2.9; CAI with regular male partners: aOR = 1.8, 95%CI = 1.1–3.0) and suffer from symptoms of depression (aOR = 2.8, 95%CI = 1.7–4.5) (each P < 0.05) (Table 2).

**Table 2:** Baseline characteristics of MSM who were victims of IPV in the past 3 months (N = 476).

Factors	N(%)	No. of IPV victims in P3M (%)	Univariate analysis		Multivariate analysis	
			Crude OR (95%CI)	P value	Ajust OR (95%CI)	P value
Ethnic						
Han	400(84.0)	69(17.2)	ref.	0.065	ref.	0.065
Non-Han	76(16.0)	20(26.3)	0.6(0.3 to 1.0)		1.7(1.0 to 3.1)	
Residence in Liaoning Province						
Yes	374(78.6)	67(17.9)	ref.	0.402	—	—
No	102(21.4)	22(21.6)	1.3(0.7 to 2.2)			
Educational level						
College and above	189(39.7)	26(13.8)	ref.	0.026	ref.	0.021
Senior high school and below	287(60.3)	63(22.0)	1.8(1.1 to 2.9)		1.8(1.1 to 3.0)	
Main venue to seek male sex partners						
Park/public bath/bars/dance halls	161(33.8)	21(13.0)	ref.	0.025	ref.	0.002
Internet	315(66.2)	68(21.6)	1.8(1.1 to 3.1)		3.0(1.5 to 6.0)	
Number of casual male partners in the P3M						
<2	292(61.3)	56(19.2)	ref.	0.735	—	—
≥2	184(38.7)	33(17.9)	0.9(0.6 to 1.5)			
Ever CRAI with male partners in the P3M						
No	370(77.7)	64(17.3)	ref.	0.145	ref.	0.149
Yes	106(22.3)	25(23.6)	1.5(0.9 to 2.4)		1.5(0.9 to 2.5)	
Ever CIAI with male partners in the P3M						
No	325(68.3)	51(15.7)	ref.	0.013	ref.	0.014
Yes	151(31.7)	38(25.2)	1.8(1.1 to 2.8)		1.8(1.1 to 2.9)	
Ever CAI with regular male partners in the P3M						
No	349(73.5)	56(16.0)	ref.	0.013	ref.	0.015
Yes	126(26.5)	33(26.2)	1.9(1.1 to 3.0)		1.8(1.1 to 3.0)	
Ever CAI with casual male partners in the P3M						
No	380(79.8)	68(17.9)	ref.	0.372	—	—
Yes	96(20.2)	21(21.9)	1.3(0.7 to 2.2)			
Recreational drug use in the P3M*						
No	427(89.7)	77(18.0)	ref.	0.275	—	—
Yes	49(10.3)	12(24.5)	1.5(0.7 to 3.0)			
Syphilis						
No	418(87.8)	80(19.1)	ref.	0.758	—	—
Yes	58(12.2)	9(15.5)	1.3(0.6 to 2.7)			
Depression (CES-D score)						
<16	299(62.8)	38(12.7)	ref.	<0.001	ref.	<0.001
≥16	177(37.2)	51(37.2)	2.8(1.7 to 4.5)		2.8(1.7 to 4.5)	

Abbreviations: PY = person-years, CI = confidence interval, OR = odds ratio, P3M = past 3 months, CAI = condomless anal intercourse, CRAI = condomless receptive anal intercourse; CIAI = condomless insertive anal intercourse.

Variables with p ≤ 0.2 in univariate analysis were entered in multivariate logistic regression model, and only variables with p < 0.05 were kept in the last model. Factors adjusted in multivariate logistic regression model included age, monthly income and marital status.

*Recreational drug included rush (poppers or alkyl nitrites), ice, amphetamines, MDMA (“ecstasy”), tramadol, and ketamine.

### HIV seroconversion and its qualitative correlation with IPV and other factors

A total of 476 MSM were recruited in this cohort, of which 437 (91.8%) were retained. Sixteen had HIV seroconversion events during a 299.5 person-year (PY) follow-up, with a calculated total incidence of 5.3 (95%CI: 3.1–8.5) /100 PY. The HIV incidence was 11.3 (95%CI: 4.7–21.9) /100 PY in MSM who had been victims of IPV in the P3M compared to 3.8 (95%CI: 1.7–7.1)/100 PY in those MSM who had not experienced IPV.

**Figure 2:**
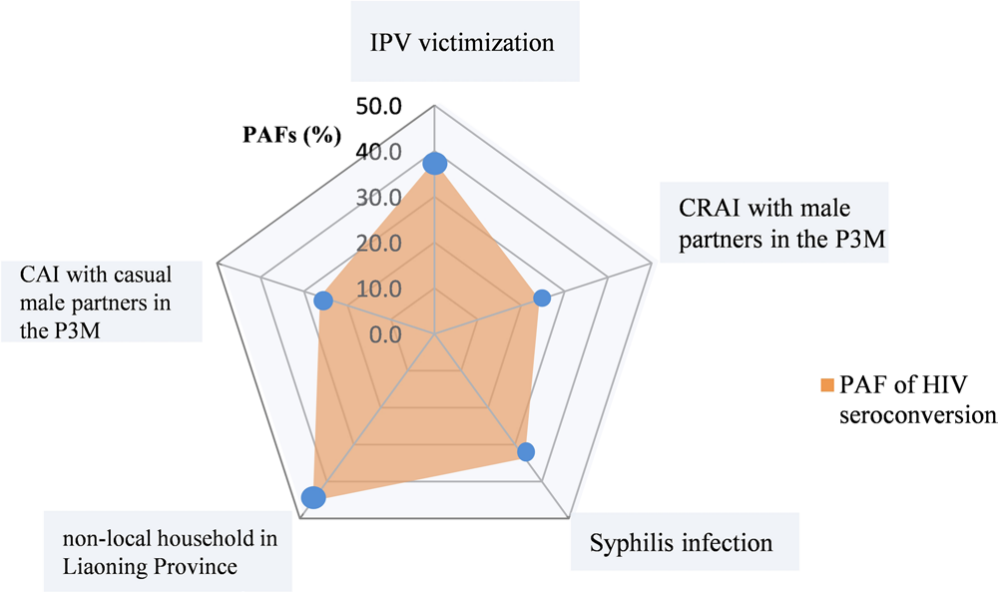
Population attributable fraction of risk factors for HIV seroconversion among Shenyang MSM.

Table 3 shows the risk factors associated with HIV seroconversion in a multivariate Cox analysis after controlling for background variables including age, education level, monthly income and marital status. As shown in the radar chart (Fig. 2), our multivariate analysis showed that individuals who had been subjected to IPV in the P3M were more likely to seroconvert to HIV (aHR = 4.1, 95%CI = 1.5–11.6), with a PAF of 37.9%, a value that was higher than that of most other behavioral risk factors. In addition to IPV, we also identified the following as risk factors for HIV seroconversion (each p < 0.05): non-local residence in Liaoning province (aHR = 3.4, 95%CI = 1.4–10.5; PAF = 45.2%), CAI with casual male partners in the P3M (aHR = 3.8, 95%CI = 1.2–3.2; PAF = 26.3%), CRAI with male partners in the P3M (aHR = 3.1, 95%CI = 1.1–9.3; PAF = 24.2%), and syphilis infection (aHR = 4.7, 95%CI = 1.6–13.5; PAF = 33.7%). Furthermore, having had ≥2 casual male partners had a marginally significant association with HIV seroconversion (aHR = 2.9, 95%CI = 1.0–8.4, p = 0.051). There was no significant difference between specific types of IPV (i.e., physical violence, or psychological violence and sexual violence) (P > 0.05) (data not shown).

**Table 3:** Univariate and multivariate COX regression with time-varying covariates and population attributable fraction analysis for risk factors of HIV seroconversion in participating MSM (N = 437).

Factors (No. of followed-up N)	No. of HIV seroconversions	Observed person-years	Incidence per 100 PY (95%CI)	Univariate		Multivariate^a^		PAF(95%CI)^b^
				Crude HR(95%CI)	*P* value	Adjust HR (95%CI)	*P* value	
Residence in Liaoning Province								
Yes (n = 343)	8	232.2	3.4(1.5 to 6.7)	ref.	0.013	ref.	0.004	45.2(0.1 to 70.0)
No (n = 94)	8	67.3	11.9(5.3 to 22.2)	3.4(1.3 to 9.2)		3.9(1.4 to 10.5)		
Number of casual male partners in the P3M								
<2 (n = 289)	7	208.1	3.4(1.4 to 6.8)	ref.	0.044	ref.	0.051	—
≥2 (n = 148)	9	91.4	9.9(4.6 to 17.9)	2.8(1.0 to 7.5)		2.9(1.0 to 8.4)		
Ever CRAI with male partners in the P3M								
No (n = 362)	11	254.9	4.3(2.2 to 7.6)	ref.	0.089	ref.	0.041	24.2(−11.3 to 48.5)
Yes (n = 75)	5	44.6	11.1(3.7 to 24.1)	2.5(0.9 to 7.2)		3.1(1.1 to 9.3)		
Ever CIAI with male partners in the P3M								
No (n = 298)	8	206.7	3.9(1.6 to 7.5)	ref.	0.103	ref.	0.335	—
Yes (n = 139)	8	92.8	8.6(3.7 to 16.2)	2.3(0.8 to 6.0)		1.8(0.6 to 5.5)		
Ever CAI with casual male partners in the P3M								
No (n = 364)	10	261.2	3.8(1.9 to 6.9)	ref.	0.010	ref.	0.028	26.3(−9.5 to 50.4)
Yes (n = 73)	6	38.3	15.8(6.0 to 31.3)	3.8(1.4 to 10.5)		3.8(1.2 to 12.5)		
Recreational drug use in the P3M^*^								
No (n = 390)	12	270.8	4.4(2.3 to 7.6)	ref.	0.050	ref.	0.204	—
Yes (n = 47)	4	28.7	13.8(3.9 to 31.7)	3.1(1.0 to 9.7)		2.9(0.6 to 14.9)		
Syphilis								
No (n = 274)	10	260.2	3.8(1.8 to 7.0)	ref.	0.011	ref.	0.010	33.7(−3.2 to 57.5)
Yes (n = 163)	6	39.3	15.3(5.6 to 33.5)	3.8(1.4–10.4)		4.7(1.6–13.5)		
Victimization of IPV in the P3M								
No (n = 351)	9	237.9	3.8(1.7 to 7.1)	ref.	0.026	ref.	0.007	37.9(−3.8 to 62.9)
Yes (n = 86)	7	61.6	11.3(4.7 to 21.9)	3.1(1.1 to 8.2)		4.1(1.5 to 11.6)		

Abbreviations: PY = person-years, CI = confidence interval, HR = hazard ratio, PAF = population attributable fraction, P3M = past 3 months, CAI = condomless anal intercourse, CRAI = condomless receptive anal intercourse; CIAI = condomless insertive anal intercourse. ^a^Variables (p ≤ 0.20) was calculated by multivariate analysis after adjusting for age, marital status, educational level, and average month income.

^b^Variables (p ≤ 0.05) was calculated by population attributable fraction. ^*^Recreational drug included rush (poppers or alkyl nitrites), ice, amphetamines, MDMA (“ecstasy”), tramadol, and ketamine.

## Discussion

As far as we are aware, this is the first prospective cohort study to explore the causal pathway between a recent IPV experience and HIV incidence among the MSM population. Our results suggest that being a recent victim of IPV was an independent determinant of HIV seroconversion among MSM in China (aHR = 4.1). Additionally, IPV had a higher PAF for HIV seroconversion (37.9%) than other traditional behavioral risk factors, such as CAI with casual male partners (26.3%) and CRAI with male partners (24.2%). Thus, our results confirm the findings of previous cross-sectional surveys^20,24,25^ and add to the existing literature by quantitatively characterizing and comparing the PAFs of IPV and other traditional behavioral risk factors for HIV seroconversion. This is important because if we want to control the spread of the HIV epidemic among MSM by adopting a comprehensive intervention strategy, this intervention must address IPV factors in male-to-male relationships.

This cohort study supports a causal pathway from recent IPV to new HIV infection. The relationship presented in this study is coherent. To control for potential time-dependent confounders, we selected a relatively shorter follow-up interval or recall period to define partner violence, captured exposure changes during follow-up, and performed a Cox model to analyze IPV and other behaviors as time-dependent covariates in order to account for time-dependent confounding. We demonstrate that being a recent victim of IPV during the follow-up period was an important contributor to the increased incidence of HIV among MSM. This is in accordance with a previous finding that experiencing IPV during one’s lifetime was associated with a subsequent HIV infection^26^. It also confirms that IPV is an important predictor of HIV incidence, with both short-term and long-term effects, and more emphasis needs to be put on mitigating its negative impact on the HIV epidemic in the MSM population.

Moreover, our research further quantitatively assessed and compared the effects of IPV and other predictors of the burden of HIV incidence in MSM. A total of 37.9% of incident HIV infection could be attributed to recent IPV victimization, which is higher than the corresponding value estimated in heterosexual women from high prevalence areas of both HIV and IPV (South Africa: PAF = 11.9%, Uganda: PAF = 22.2%)^12,28^. This implies that interventions aimed at effectively controlling violence in the MSM population would achieve a better reduction in the incidence of HIV than in heterosexual populations. Compared with the traditional behavioral risk factors detected in this study, we also found that a higher PAF was associated with recent victimization of IPV. This indicates that violence has a greater impact on HIV seroconversion than traditional behavioral predictors in MSM. These findings illustrate that IPV prevention may be a highly cost-effective strategy for controlling the ever-increasing HIV epidemic in MSM, and a comprehensive HIV intervention strategy that includes IPV evaluation and intervention must be established to control the spread of HIV in the MSM community.

In addition to IPV, traditional predictors such as CAI, male sexual partner concurrency and syphilis infection remained important contributors to the incidence of HIV in our MSM subjects. Although other studies have confirmed their negative impact on the HIV epidemic^29–31^, our data also highlight behavioral interventions that would be the most beneficial in the MSM population to curb the spread of HIV. Our study also observed a positive link between migrant MSM and the incidence of HIV, i.e., migrants may lack social support and their MSM subpopulation lacks access to the healthcare system, making them a high risk group^32,33^. It will be important to determine whether this subpopulation also suffers from a higher rate of IPV in the future.

At baseline, we found that about one-fifth of MSM subjects in this study were victims of IPV in the past 3 months, with a prevalence of physical IPV of 10.0%, psychological IPV of 13.7% and sexual IPV of 9.5%. These values are somewhat higher than what were previously reported with respect to recent IPV experiences among MSM. For example, the reported prevalence of experiencing either physical or sexual IPV was only 7.3% among American MSM couples in the past 3 months^34^. One six-country multicenter study on MSM revealed the prevalence of sexual IPV in the past year ranged from 2.5% to 4.5%^35^. Although there is a lack of available comparison data on recent IPV experiences in China, a previous survey of MSM in Shanghai reported that approximately 40% MSM had experienced some form of IPV during their lifetime^15,16^. These results seem to indicate that MSM in China experience a higher level of violence, and therefore public health departments should cooperate with the public security department to strengthen IPV screening and develop professional skills to collect IPV information from this vulnerable population.

Furthermore, our findings showed that victims of IPV are more likely to be involved in sexual risk-taking and to suffer from depression. These could be important contributors to the causal pathway from IPV to HIV infection identified in this study. In our MSM cohort, IPV victims had a lower education level, which is similar to what was observed among heterosexual women and MSM in Western countries^36–38^. A lower education level may be a barrier to social, economic, and healthcare resources, leading to an elevated vulnerability to IPV as well as HIV infection^36,39–41^. Additionally, our findings are consistent with peer studies that show that victims are more likely to be engaging in CAI^15,22,42,43^. We also found that IPV victims seemed more likely to find their sexual partners over the internet. MSM who were recruited via the internet are more likely to encourage high-risk sexual behaviors^44–46^. These risky behaviors could increase the risk of acquiring HIV among IPV victims. Moreover, our findings are consistent with previous studies from western countries that victims of IPV are more likely to suffer from symptoms of depression^18,47,48^. Men in a violent relationship who are being victimized and suffer from symptoms of depression might encounter difficulties in seeking help to end the relationship, or their turbulent relationships may make them poorly compliant with prescribed treatment. This finding could partially explain the important role of IPV as a significant contributing factor to HIV acquisition among MSM. It may also support the hypothesis that IPV health professionals that offer traditional behavioral strategies combined with psychiatric help might have a greater potential effect than those working with victims in isolation.

Although IPV was shown to be an important contributing factor to the HIV epidemic among MSM, the current support services and interventions for MSM are still inadequate^49^. For this reason, replicating this association in the context of trials capable of assessing effective prevention strategies should be a priority. First, given the higher level of risky sexual behaviors among victims of IPV, traditional behavioral intervention strategies need to incorporate IPV. The value of combined prevention strategies has been shown to be effective in the general population of Uganda^50^. Second, in recent years, many studies have shown that bio-medical prevention, in particular PrEP combined with HIV prevention, could reduce the risk of acquiring HIV among MSM and other high-risk populations^51,52^. Hence, the popular PrEP strategy may be a good candidate strategy for reducing the risk of HIV among MSM who are affected by IPV. Third, it is essential to establish and improve IPV support services for the MSM population to help IPV victims terminate a troubled relationship as soon as possible. IPV has been said to be a barrier to HIV disclosure^53^, and therefore may increase the risk of HIV transmission. Finally, it will also be necessary to change the social attitudes, beliefs, and cultural norms that support IPV^54,55^.

This study has the following strengths; it is the first prospective cohort study to examine the causal relationship between recent IPV to HIV seroconversion in the MSM population. We also quantitatively calculated the effect of IPV on HIV seroconversion by estimating the PAF, which can help public health workers to better understand the extent of IPV’s influence on HIV. Our cohort retention rate was high, owing to multiple measures taken to maintain participant compliance during follow-up. We used Cox proportional hazard models and analyzed time-dependent covariates to better understand the influence of IPV on HIV incidence among MSM.

This study also has some limitations. Since IPV is a sensitive subject for study participants, we cannot rule out the possibility of a social desirability bias through self-reporting measures. It may not be possible to generalize these results to other MSM populations in other countries or regions because the recruitment occurred only in Shenyang, China. Although we followed this cohort for a relatively long time (12-months), a longer observation period would have been beneficial to further confirm our findings. We did not collect the lifetime IPV experiences among MSM. The results of this study found a significantly higher incidence of HIV among MSM who were victims of IPV, but whether this phenomenon could be observed in the perpetrators of IPV in this population will need to be verified in future studies. Out of consideration for the direction of this study, we did not deeply explore the formation of the internal social psychology of IPV among MSM, which will be an important direction for future research.

In conclusion, in addition to conventional high-risk behaviors, being a recent victim of IPV was an important contributor to HIV seroconversion in our MSM population. IPV also seemed to increase the risk of CAI and depression. Therefore, addressing IPV should be a priority during HIV intervention. HIV prevention strategies need to become more sophisticated and should incorporate IPV screening followed by effective intervention. In the subgroup of MSM affected by IPV, it is also essential to pursue bio-medical prevention (including PrEP) in order to reduce their risk of acquiring HIV. It should be a priority to establish and improve the support services for IPV victims and to give them advice on how to terminate an unhealthy, potentially life-threatening relationship.

## Methods

### Ethic Statement

This study was approved by the Institutional Review of the First Affiliated Hospital of China Medical University, and research procedures were carried out strictly in accordance with relevant guidelines. Written informed consents were obtained from each participant prior to their interview and blood collection. To ensure privacy, every participant was uniquely assigned an identification code (PID) instead of his real name during the survey to link their questionnaire and laboratory testing results.

### Study design and enrollment

From January 1st until December 31st, 2014, an open cohort enrollment of HIV-negative MSM was implemented in Shenyang city in Northeast China, which with an estimated 140,000 MSM has the second-largest population of MSM in China after Beijing^56^. Recruitment was performed in two ways: (1) Venue-based sampling: with the help of the Shenyang Sunshine Working Group, a local MSM non-government organization (NGO), eligible MSM were recruited from suitable venues (e.g., MSM parks, bars, clubs and bathhouses); (2) Peer referrals: MSM were encouraged to recruit their MSM friends or peer male partners to take part in this survey.

Inclusion criteria included the following: (1) HIV serologic negativity; (2) biologically male; (3) 18 years of age or older; (4) self-reported admission of having anal intercourse with male partners within the past six months, and (5) were willing to participate in the cohort and provide written informed consent. Eligible MSM participants were followed-up on every three months. At both baseline and follow-up visits, questionnaire-based interviews and risk-reduction counseling were conducted by trained staff in a separate private interview room of the VCT (HIV Voluntary Counseling & Testing) clinic. Each interview lasted for an average of half an hour, and was conducted by a trained experienced interviewer with at least an undergraduate degree. To meet the psychological needs of each MSM, our interview team consisted of two male interviewers and one female interviewer. No MSM participants had received or were receiving PrEP, as there are still no PrEP guidelines in mainland China. An experienced physician then collected 10 ml of venous blood for HIV and syphilis testing. Those subjects who tested positive for HIV or syphilis were referred to clinics or hospitals to receive appropriate treatment and subsequent medical care. A local MSM non-government organization (Shenyang Sunshine Working Group), and other relevant departments provided counseling and referral services at the study site for MSM subjects who were affected by IPV.

### Maintenance of follow-up

To maximize retention, every participant was asked to provide at least two current forms of contact information, including mobile phone numbers and internet-based chat services (WeChat or Tencent QQ), which could be used as reminders for up-coming and missed appointments. Specially dedicated research staff were in charge of ensuring follow-up. Reminder calls were made 1 to 2 weeks before the follow-up day. When a participant missed a follow-up day, staff would call the participant to reschedule the next appointment as soon as possible. If participants could not be reached by phone, WeChat or Tencent QQ messages and peer acquaintances would be used to contact the participants. We offered progressive incentives to further encourage participants to continue to follow-up: participants who attended the baseline visit were given 30 RMB (approximately US$4.34) in cash; those who completed the second visit were given 40 RMB (approximately US$5.79) in cash; those who completed the third visit and beyond were given 50 RMB (approximately US$7.24) in cash.

### Data collection

Face-to-face interviews using structured questionnaires were performed to collect the participants’ demographic, behavioral, and IPV information. Depression assessments were performed at baseline and at each 3-month follow-up visit.

### Socio-demographic and behavioral variables

Participants were asked to report their socio-demographic information (e.g., residence location, educational level, monthly income, and marital status), sexual risk behavior in the P3M (e.g., multiple male sexual partners, condom use with different partners and role) and substance use in the P3M [e.g., rush (poppers or alkyl nitrites), ice, ketamine, amphetamines, MDMA (“ecstasy”), and tramadol].

### Intimate partner violence

To obtain accurate estimates of current victimization among male-to-male relationships, our definition of an IPV experience was the same as the one issued by CDC^57^. Asking behaviorally specific questions encouraged greater disclosure. The respondents could identify themselves as victims and describe how their partners treated them. Respondents were asked to report “unwanted partner violence” (in order to eliminate participants who were masochists) from a male-to-male sexual partner in past three months based on a series of questions. Participants who responded with “yes” to any of these questions were defined as having been a victim of IPV.

### Depression

The study’s approach to measuring symptoms of depression was assessed by the Center for Epidemiologic Studies Depression Scale. CES-D scores ranged from 0 to 60, with 20 items that described either somewhat elevated or very elevated levels of depression symptoms. The symptoms of depression were quantified by the total score (score of 0–15: no depression; score greater than 15: may have depression)^58^.

### Laboratory tests

Blood specimens were collected from each participant for diagnosing HIV and syphilis. HIV serostatus was evaluated with an enzyme-linked immunosorbent assay (ELISA) (InTec Products Company, Xiamen, China), and confirmed with an HIV-1/2 western blot (HIV Blot 2.2 WBTM, Genelabs Diagnostics, Singapore). We selected syphilis, a common STI disease among MSM, as a biologic marker to reflect high-risk behavior, mainly because syphilis detection sampling is more convenient and is highly accepted. Syphilis infection status was determined using the rapid plasma reagin (RPR) test (Shanghai Rongsheng, Shanghai, China), and confirmed with the Treponemapallidum particle assay (TPPA) (Fujirebio Inc, Tokyo, Japan). Only samples that yielded positive results in both tests were considered syphilis-positive (i.e., recently and currently infected with syphilis) when the seroprevalence of syphilis was calculated. Otherwise a negative result was reported^59^.

### Statistical analysis

In our analysis, an HIV seroconversion event was assumed to have occurred at the midpoint between the time of a new HIV diagnosis and the time of the most recent previous HIV-negative visit. Unconditional logistic regression models were performed to examine baseline factors associated with the prevalence of IPV. Factors with P < 0.20 in the univariate analysis were included as logistic variables, and those with P < 0.05 were retained in the final multiple regression model.

Time-dependent Cox proportional-hazards regression models adjusting for the effects of exposure changes on outcome were estimated with adjusted hazard ratios and 95% confidence intervals (CI) as a measure of the relative risk of HIV seroconversion. Factors with P < 0.20 in the univariate analysis were included in the multivariate Cox model, with adjustments for potential confounders such as age, monthly income, education level, and marital status. An important feature of this model is that it includes time-dependent covariates (including IPV and other behavioral factors as well as syphilis/depression status) in the risk set at each observation time point according to their covariate status, which can change at different time intervals.

To specifically assess the effects of IPV on the incidence of HIV, variables kept in the multivariate Cox regression model were further analyzed by PAF and associated 95% CIs, which reflect the fraction of HIV incidence in the whole population. Here, population is defined as the cohort that is associated with the exposure of IPV and other relative factors. This was further computed based on the aHR using the standard formula [p(aHR – 1)/aHR]^60^. STATA (StataCorp LP) Version 13.0 was employed for data analysis. A two-tailed P < 0.05 was considered statistically significant.
